# Case of Hæmorrhage of the Liver

**Published:** 1845-01

**Authors:** Jas. Abercrombie

**Affiliations:** Cape of Good Hope


*Case of Hæmorrhage of the Liver.* By Jas. Abercrombie, M.D. Cape of Good Hope.—The patient, a lady aged 35, died on the fourth day after delivery. On examination after death, there was found on the anterior and superior surfaces of the liver, a large sac, which burst on attempting to remove the organ, and discharged about two pounds of blood, fluid and coagulated; it was found to have escaped from a branch of the venæ portæ, and the sac was formed by the peritoneum. The organ itself had throughout a mottled appearance and was unusually soft. The uterus was in a perfectly sound state, as were all the other viscera both of the pelvis and abdomen.—*London and Edinburg Monthly Journal.*

